# Activating electrochemical catalytic activity of bio-palladium by hybridizing with carbon nanotube as “e^−^ Bridge”

**DOI:** 10.1038/s41598-017-16880-7

**Published:** 2017-11-29

**Authors:** Hao-Yi Cheng, Ya-Nan Hou, Xu Zhang, Zhen-Ni Yang, Tiefu Xu, Ai-Jie Wang

**Affiliations:** 1 Key Laboratory of Environmental Biotechnology, Research Center for Eco-Environmental Sciences, Chinese Academy of Sciences, Beijing, 100085 China; 2 State Key Laboratory of Urban Water Resource and Environment, Harbin Institute of Technology, 73 Huanghe Road, Harbin, 150090 China; 3 Center for Microbial Ecology and Technology, Ghent University, Coupure Links 653, Ghent, B-9000 Belgium; 4 School of Civil Engineering, Heilongjiang University, Harbin, 150080 China

## Abstract

Nano metal catalysts produced by bacteria has received increasing attention owing to its environmental friendly synthesis route. However, the formed metal nanoparticles are associated with poorly conductive cells and challenged to be electrochemically applied. In this study, Palladium (Pd) nanoparticles were synthesized by *Shewanella oneidensis* MR-1. We demonstrated the limitation of palladized cells (Pd-cells) serving as electro-catalysts can be relieved by hybridizing with the conductive carbon nanotubes (Pd-cells-CNTs hybrid). Compared to the Pd-cells, the electrochemical active surface area of Pd in Pd-cells-CNTs10 (the ratio of Pd/CNTs is 1/10 w/w) were dramatically increased by 68 times to 20.44 m^2^·g^−1^. A considerable enhancement of electrocatalytic activity was further confirmed for Pd-cells-CNTs10 as indicated by a 5-fold increase of steady state current density for nitrobenzene reduction at −0.55 V vs Ag/AgCl. These results indicate that the biogenetic palladium could has been an efficient electro-catalyst but just limited due to lacking an electron transport path (e^**−**^ Bridge). This finding may also be helpful to guide the way to electrochemically use other biogenetic metal nano-materials.

## Introduction

Metal nanoparticles (NPs) synthesized by bacteria, a cost-effective and environmental friendly method, have been attracted increasing attention in the past years^1,2^. These biogenetic metals shows excellent properties in terms of catalysis^3^, disinfection^4^ and Raman analysis^5^. In the case of environmental application, biogenic palladium NPs (bio-Pd) are one of the most concerned metals, because its distinctive hydrogenation effect could substantially accelerate the reductive degradation of many recalcitrant contaminants, such as nitroaromatics, polychlorinated biphenyls and azo dyes^6–8^.

To date, the feasibility of Pd NPs production has been demonstrated in a number of bacteria, including *Shewanella* ^9^, *Geobacter* ^10^, *Desulfovibrio* ^11^, *Enterococcus* ^12^, etc. Efforts to improve the catalytic activity of bio-Pd have been made by controlling the size and distribution of Pd NPs or by introducing another metal to form Pd based bimetal NPs^13,14^. In addition, to avoid the loss of bio-Pd, magnetite was proved to be co-deposited with Pd on the cell and facilitated the its recovery^6^. These works, therefore, further strengthen the application potential of bio-Pd.

When using bio-Pd as a catalyst for the reductive degradation of contaminants, reductants, such as hydrogen, formate and borohydride, are the common involved the electron donors. These chemical reductants however are not naturally contained in the wastewater and therefore require external supply, which could raise the cost in particular for their storage and transportation. Alternatively, internal supply of reducing power through electrode is proposed as a safe and sustainable manner. This idea were previously carried out by simply coating palladized cells (Pd-cells) on the graphite and showed the enhanced electrochemical catalytic activity for the dehalogenation of diatrizoate and trichloroethylene^15,16^. However, as bio-Pd is supported by the bacteria that are poor electric conductive, the deteriorative performance after coating Pd-cells on electrode was also observed sometimes^17^. Although the carbonization of biomass could improve the conductivity, it requires additional energy input and may suffer from the aggregation of Pd NPs due to the high temperature^18,19^. Furthermore, the catalytic properties of Pd could be changed during this process due to transformation of Pd to its oxide^19,20^.

**Figure 1:**
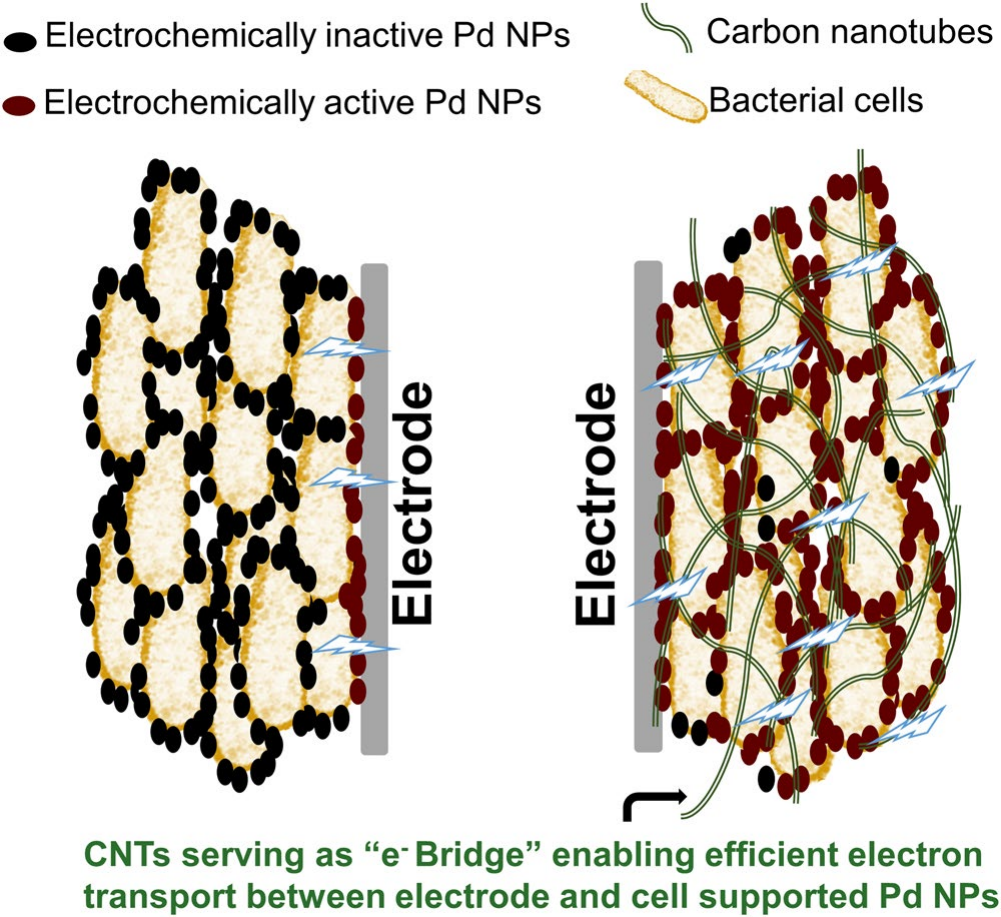
Schematic illustration of CNTs playing a role as “e^−^ Bridge” to enable the Pd NPs on bacterial cells electrochemically active.

The distribution of Pd NPs is usually sporadic on the cell surface, especially when they have relative small size. That means, when the Pd-cells are coated on the electrode, only the Pd NPs contacting the electrode are capable of receiving electrons efficiently, while the rest accounting for the majority are electrochemical inactive due to the hindrance from poorly conductive biomass. Based on this concern, we assume that Pd NPs far away from electrode surface can be activated by compositing the Pd-cells with conductive material as an electron transport path (“e^**−**^ bridge”, Fig. 1). In this way, the electrochemical catalytic activity of Pd-cells is expected to be enhanced without additional pretreatment.

In this study, Pd NPs was synthesized by a typical dissimilatory metal reduction bacterium, *Shewanella oneidensis* MR-1. Carbon nanotubes (CNTs) were chosen to serve as the “e^**−**^ Bridge” owing to the excellent conductivity^21^. The activating effect of CNTs on bio-Pd was evaluated by measuring the electrochemical active surface area of Pd (EASA_Pd_) as well as the capability of under potential hydrogen deposition (UPD H). The enhanced electrochemical catalytic activity of Pd-cells with the composition of CNTs was further verified by using nitrobenzene as a mode contaminant through cyclic voltammetry and chronoamperometry tests.

## Results

### Bio-Pd formation and characterization

**Figure 2:**
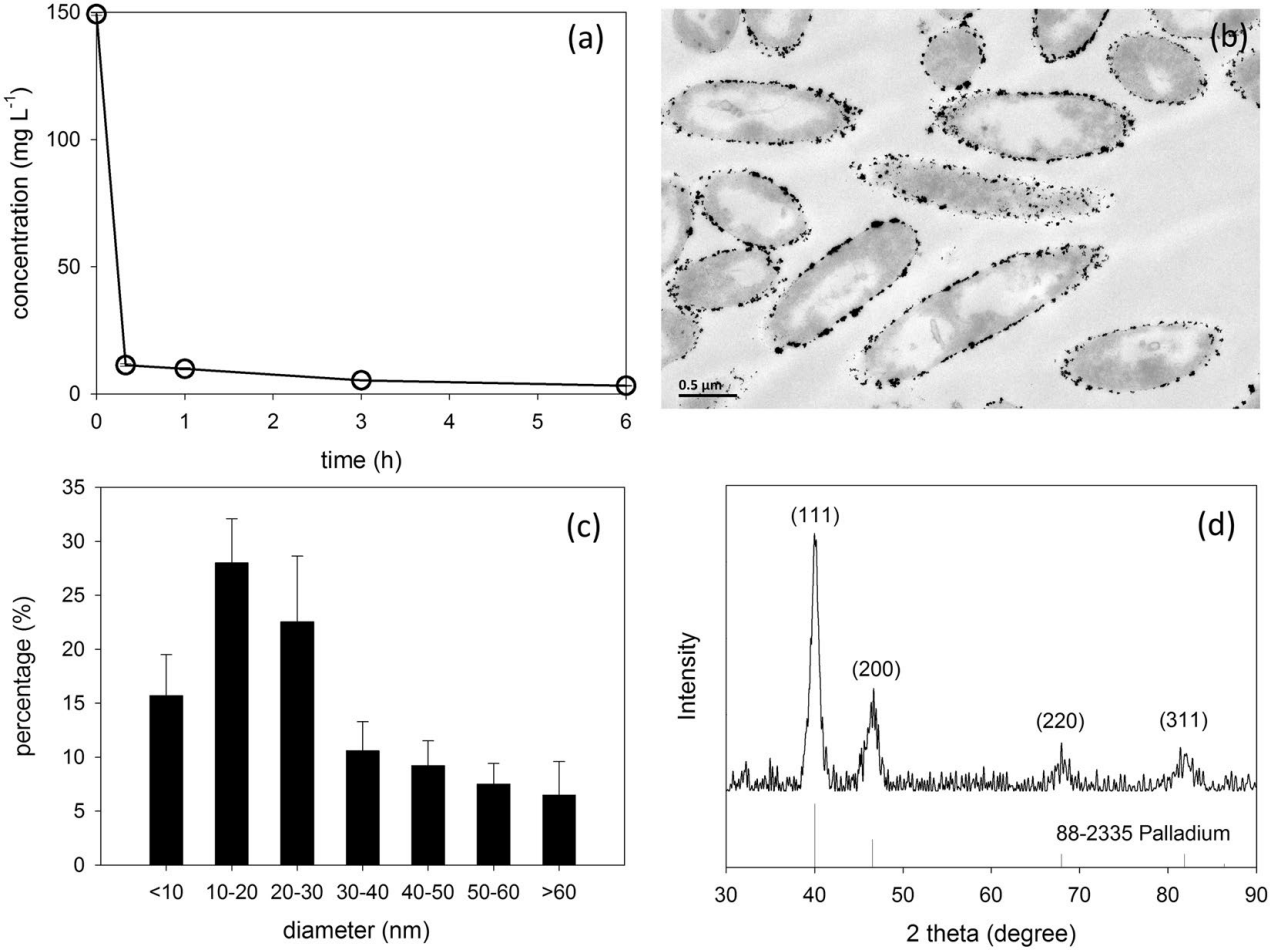
The evolution of Pd(II) concentration during the bio-Pd synthesis (**a**). TEM image of the ultrathin slice (**b**) and the XRD analysis (**d**) of the Pd-cells. Size distribution of the Pd NPs deposited on the cells surface (**c**).

After the concentrated Pd(II) adding into the formate amended *Shewanella* suspension, the color of the suspension started to change from yellow to black as expected in a few minutes. ICP analysis showed the decline of Pd(II) concentration is fast in the first 30 min and then became smooth. Within 6 hours, over 95% of the Pd(II) was reduced (Fig. 2a). According to the observation of ultrathin section by TEM, a large number of nanoparticles (NPs) with sizes centered on 10~30 nm were formed on the cells surface (Figs 2b and c). These NPs were further confirmed as Pd(0) according to the X-ray diffraction (XRD) analysis (Fig. 2d). The strongest reflection was observed at 2θ = 39.8° (111), indicating the main crystalline of Pd NPs follows the structure of face centered cubic.

### Evidences of the composited CNTs acting as “e^**−**^ Bridge” for bio-Pd

**Figure 3:**
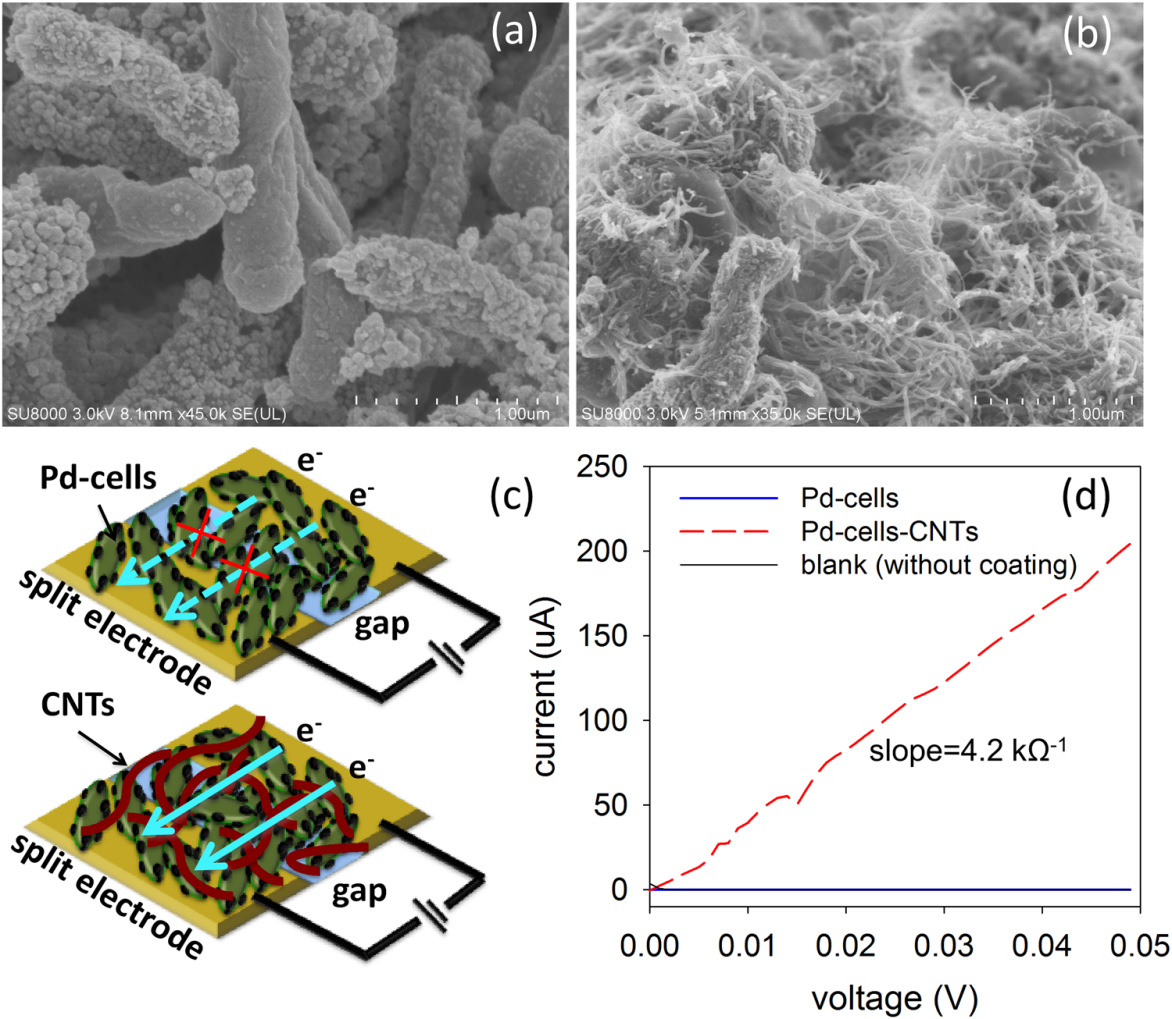
SEM images of Pd-cells (**a**) Pd-cells-CNTs hybrid (**b**). Schematic illustration of the conductance measurement experiment which highlighted the improvement of electron transport via CNTs network in the Pd-cells-CNTs hybrid (**c**). I-V curves obtained by applying the gradually increased voltage across the split electrodes coated by Pd-cells and Pd-cells-CNTs hybrid, or without coating (**d**).

As observed in the SEM images, the Pd-cells are shown to be deposited by a lot of sporadically distributed Pd NPs (Fig. 3a), which is consistence with that reported in other works^8,22^. After the formation of Pd-cells-CNTs hybrids, the Pd-cells are shown to be trapped into the CNTs network and connected to each other through the CNTs (Fig. 3b).

To understand if these CNTs could act as the “e^**−**^ Bridge” in Pd-cells-CNTs hybrid, Pd-cells and the hybrid were coated on the split gold electrodes with the gap width of ~100 μm (Fig. 3c). As shown in Fig. 3d, the conductance of Pd cells that extracted from slope of i-v curves was almost the same as that of the control (without coating), which was close to zero. This result supports our hypothesis that the electron transfer across the Pd-cells is inefficient. Considering the Pd-cells-CNTs hybrid, current obviously increased with voltage across the split electrode, where the conductance is calculated as 4.2 kΩ^−1^. The dramatic enhancement of conductance in the hybrid clearly indicated CNTs can facilitate the electron transport throughout the hybrid.

**Figure 4:**
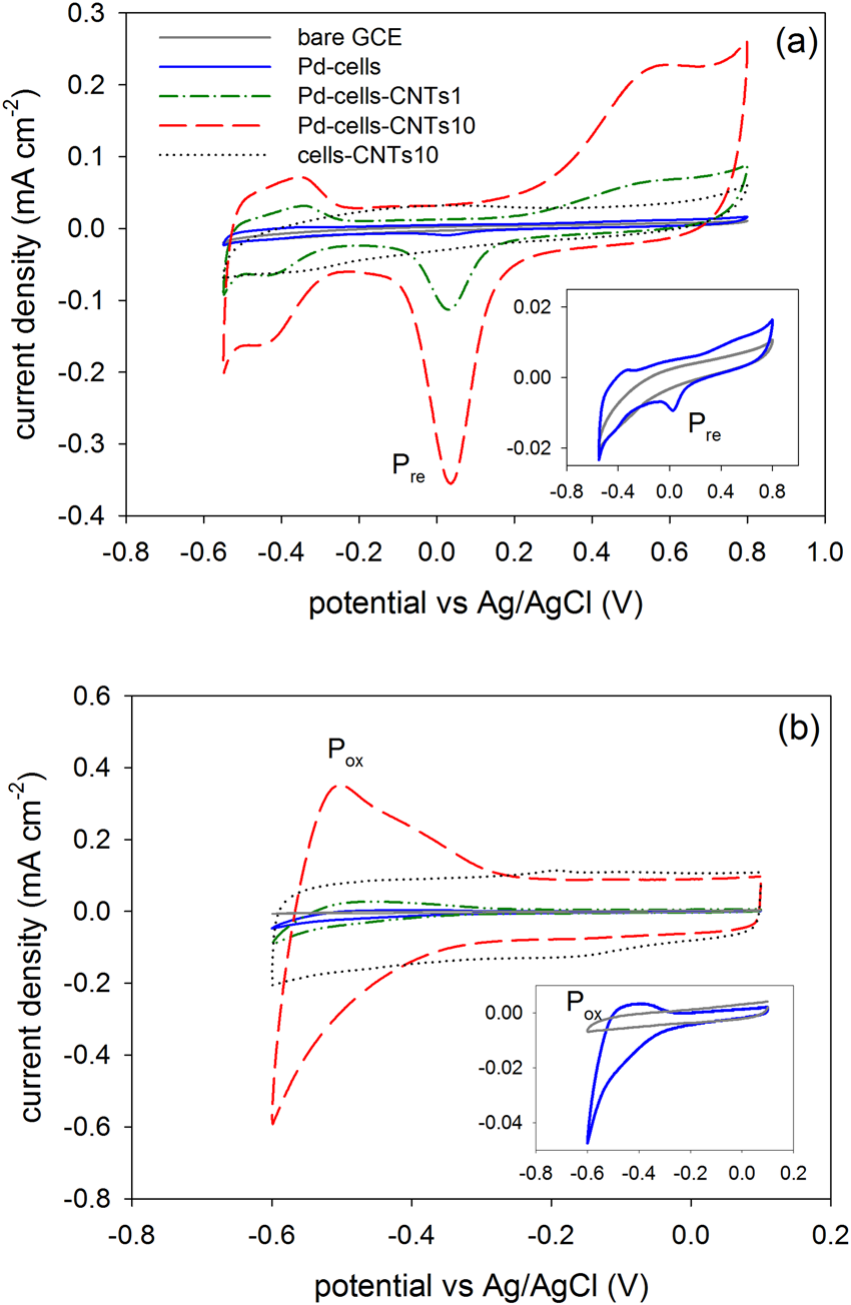
Cyclic voltammograms of GCE and that coated with Pd-cells, Cells-CNTs hybrid and Pd-cells-CNTs hybrids in 50 mM phosphate buffer solution that were used to estimate the EASA_Pd_ (**a**) and UPD H (**b**). The scan rate was 10 mV·s^−1^.

CV was employed to estimate the electrochemical active surface area of Pd (EASA_Pd_). As shown in Fig. 4a, a reduction peak (P_re_) at 0.045 V appeared in Pd-cells CV, which attributed to the reduction of Pd oxide formed during the positive scan^23^. Accordingly, the EASA_Pd_ was calculated as 0.29 m^2^·g^−1^. After hybridizing with CNTs at equal amount to the Pd, the peak current density of P_re_ in Pd-cells-CNTs1 increased more than one order of magnitude. The corresponding EASA_Pd_ increased to 6.01 m^2^·g^−1^, which was about 20 times higher than that obtained in Pd-cells. Since the Pd loading amounts for Pd-cells and the hybrid were controlled identically, the increase of EASA_Pd_ in the hybrid clearly suggests that the CNTs can play the role as an “e^**−**^ Bridge” to activate those Pd NPs that are not electrically connected to the electrode. With more CNTs composited in the hybrid (Pd-cells-CNTs10), a further increase of the peak current density by 3.4 times was observed at P_re_. Correspondingly, the EASA_Pd_ enlarged to 20.44 m^2^·g^−1^. Here, the further increase of EASA_Pd_ is likely because the higher amount of CNTs provides more connections and enables more cell supported Pd NPs to be electrochemical active.

Additional evidence to support CNTs serving as “e^**−**^ Bridge” resulted from the unique under potential hydrogen deposition (UPD H) property of Pd. As shown in Fig. 4b, the occurrence of UPD H was indicated by the obvious increase of cathodic current before the theoretical hydrogen evolution potential (−0.61 V vs Ag/AgCl, pH = 7). The deposited hydrogen was then re-oxidized during anodic process. The oxidation peak (P_ox_) can reflect the amount of deposited hydrogen by integrating the area of P_ox_. Notably, as the UPD H is an electrochemical process, it can only occur at the Pd that electric connected to the electrode. Besides, since the coated Pd-cells or hybrids had the same amount of Pd that came from the same source, the area of P_ox_ should correlate to the amount of the electrochemical active Pd. Compared to Pd-cells, the area of P_ox_ in Pd-cells-CNTs1 and Pd-cells-CNTs10 were enlarged by 9.5 and 105 times, respectively (Fig. 4c). Because the P_ox_ in the hybrid without Pd (cells-CNTs10) was negligible, it excluded the contribution of CNTs themselves to the enhancement of UPD H and confirmed the role of CNTs played as an “e^**−**^ Bridge”.

### Enhancement of electro-catalytic activity of bio-Pd by hybridizing with CNTs: NB reduction as a case

**Figure 5:**
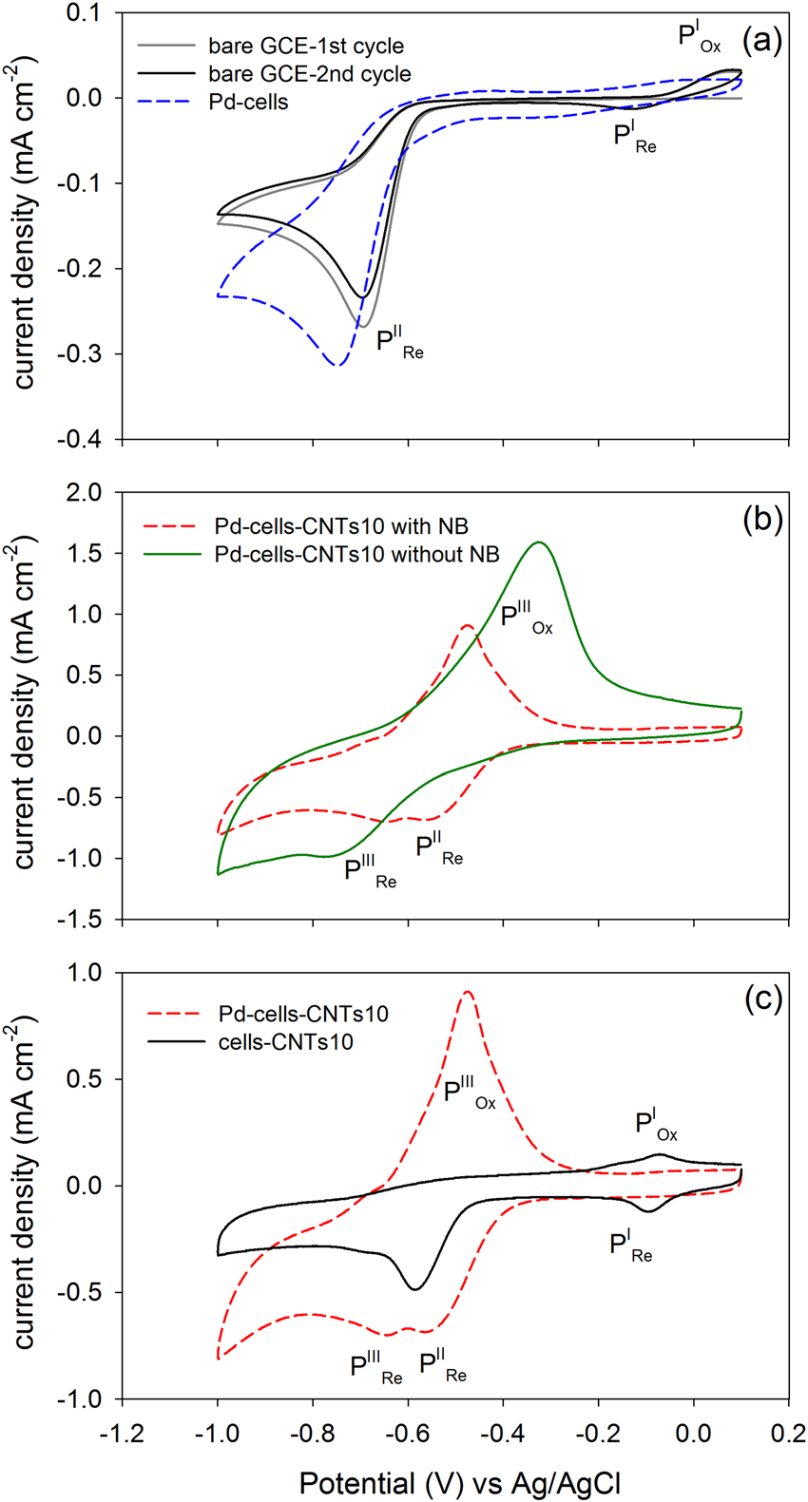
CVs of bare GCE and ones that coated with Pd-cells, Pd-cells-CNTs10 and cells-CNTs10 in NB (0.5 mM) amended phosphate buffer solution (50 mM) (**a**,**c**). CVs of GCE coated with Pd-cells-CNTs10 in 50 mM phosphate buffer solution with and without NB (0.5 mM) (**b**). Except the CV of bare GCE, CVs of other samples were shown as the 2^nd^ cycle. All CVs were performed at the scan rate of 10 mV·s^−1^.

CV test showed NB reduction at bare GCE started when potential negative sweeping to −0.548 V (Fig. 5a). During backward scan, an oxidation peak (P^I^_Ox_) appeared at 0.03 V which resulted in a new reduction peak (P^I^_Re_) in the second negative scan. This pair of peaks has been well studied as the transformation between phenylhydroxylamine (PHA) and nitrosobenzene (NOB), the intermediates during NB reduction to aniline (AN). After coating with Pd-cell, NB reduction peak (P^II^_Re_) increased by about 35% (comparison was according to the second scan), indicating improvement of NB reduction rate. Regarding Pd-cell-CNTs hybrid, the CV profile was shown differently with two reduction peaks (P^II^_Re_ at −0.520 V and P^III^_Re_ at −0.635 V) and one oxidation peak (P^III^_Ox_ at −0.485 V) (Fig. 5b). As the control, CV of the hybrid was also performed under the condition without NB, showing one reduction peak at –0.745 V and one oxidation peak at −0.335 V which could be attributed to the electrochemical hydrogen absorption/adsorption and desorption^8^. By comparing the CV with and without NB (Fig. 5b), the faradic current coming from P^II^_Re_ was only observed in the presence of NB, indicating the P^II^_Re_ associated with the electrochemical NB reduction. Compared to Pd-cells CV, P^II^_Re_ in the CV of Pd-cells-CNTs10 showed 2.3-fold increase of peak current density and positively shifted ~0.04 V, indicating the enhancement of the electrochemical catalytic activity for NB reduction.

Another CV control test was carried out by using the electrode coated with cells-CNTs hybrid (Fig. 5c). The CV profile of cells-CNTs10 is similar as that observed in bare GCE test but quite different from that in Pd-cells-CNTs10 CV. Compared to the CV of bare GCE, electrode coated with cells-CNTs generated higher peak current density in NB reduction (P^II^_Re_), which can be explained as CNTs providing larger surface area for the reaction^24^. However, compared to the Pd-cells-CNTs10, NB reduction peak (P^II^_Re_) in cell-CNTs10 was smaller and appeared from more negative potential. This observation indicates the enhancement of NB reduction in Pd-cells-CNTs hybrid is not simply due to the increase of surface area contributed by CNTs, but also results from more cell supported Pd NPs becoming electrochemically active through CNTs serving as the “e^**−**^ Bridge”. To be noted, the peaks (P^I^_Ox_ and P^I^_Re_) correlated to the redox reaction between PHA and NOB almost disappeared in the CV of Pd-cell-CNTs10 as compared to that of cells-CNTs10. This finding implied the reduction of NB catalyzed by Pd-cell-CNTs hybrid could generate more reduced product (i.e. aniline), likely because the electrochemical adsorbed/absorbed hydrogen at/into the Pd NPs participated in the process of NB reduction and further reduced the intermediates.

**Figure 6:**
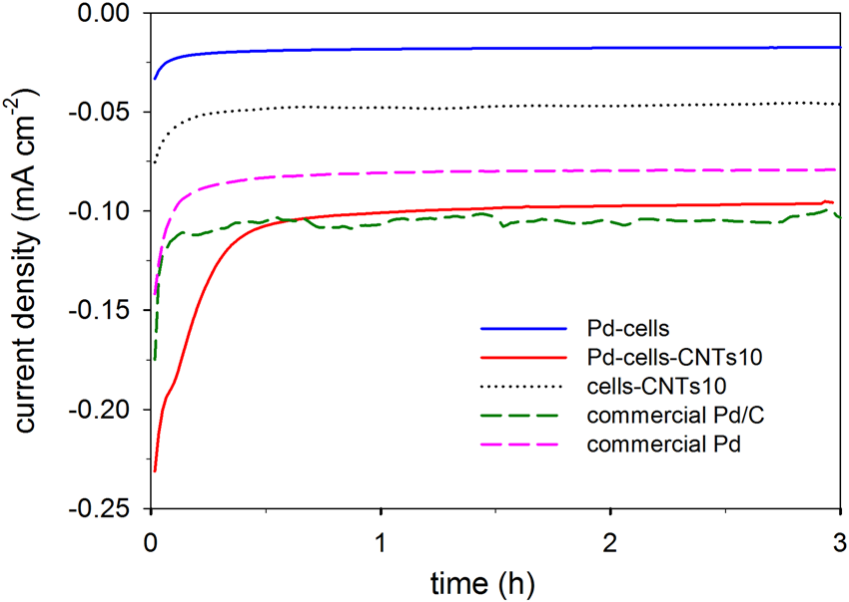
Chronoamperomograms of GCE coated with Pd-cells, Pd-cells-CNTs10, cells-CNTs10 and commercial Pd catalyst in NB (0.5 mM) amended phosphate buffer solution (50 mM).

Chronoamperometry tests were further carried out to show the electrochemical NB reduction at steady state (Fig. 6). Herein, the experiment was conducted by applying potential at −0.55 V to minimize the occurrence of hydrogen evolution. In this way, the electrons for NB reduction can be controlled to be delivered through a conductive path but not via the molecular hydrogen as the electron mediator. As shown in Fig. 6, with the CNTs composition, the steady-state current density for NB reduction increases 5 times from ca. 0.02 mA·cm^−2^ (Pd-cells) to ca. 0.1 mA·cm^−2^ (Pd-cell-CNTs10). Since the steady-state current density in cells-CNTs is only ca. 0.05 mA·cm^−2^, the substantial improvement for NB reduction in the Pd-cell-CNTs as compared to the Pd-cells can be confirmed mainly due to the activation of cell supported Pd NPs by CNTs as the “e^**−**^ Bridge”. Compared to the current density of the commercial catalysts, the performance of Pd-cell-CNTs for NB reduction was within range of the Pd/C catalyst while superior to the Pd catalyst with about 20% increase.

## Discussion

Bacterial synthesized metal NPs are generally considered to be the poor electrochemical catalysts because the co-existed less-conductive bio-material could hinder the electron transfer between the electrode and metal NPs. Two hypotheses for this issue were proposed before: (i) metal NPs are sporadically distributed in/on the cells so that a connected electrical conductive path cannot be created^8,17^; (ii) metal NPs are surrounded with some bio-produced organic materials which play a role of an insulating layer^25^. In this study, the addition of CNTs into Pd-cells has been found to dramatically improve the electron delivery from the electrode to the Pd NPs. That meant the surface of Pd NPs was able to expose to the CNTs and the hypothesis related to insulating layer was not supported by this work. Since the property of the possible organic layer has not been well studied, it is still unclear whether the surface exposure of Pd NPs is due to the overestimation of the coating or the undesigned removal of the layer during the voltex mixing of the CNTs and the Pd-cells.

In order to overcome the poor conductivity of biomaterial-metal NPs complex, strategy by using high temperature to carbonize the biomaterials has been extensively employed^26–29^. However, this method requires additional energy consumption and may lead to the aggregation of NPs, resulting in the decease of specific surface area and decline the catalytic activity^19,26^. Additionally, to avoid carbonized biomass blocking the active sites of metal NPs, oxygen had to be introduced to remove excessive carbon^26^, which could increase the chance of forming less-conductive metal oxide layer and may attenuate the electrochemical activity^20,30^. In addition to carbonization, bio-material removal by chemical cleaning was also proposed^25^. However, this approach involved in using strong base and required long treatment time up to half year. Compared to the existing methods, hybridizing with conductive CNTs as reported here is obviously much simpler and more environmental friendly without neither high energy input nor using hazardous chemicals. More importantly, this method does not change the property of metal NPs and remains the benefit of biomass that keeps the stable of NPs as well as prevents its loss^22,31^.

Directly compositing Pd-cells with other conductive materials, such as carbon granule and activated carbon powders (ACP), were reported but with less satisfied electrocatalytic activities^17,28^. With regard to carbon granules, although they can form a conductive matrix themselves, the space volume among the granules could reach the scale of millimeter, which seems too large to allow the abundant connections of the granule surface to cell supported Pd NPs. Therefore, the graphite granules more likely just played a role as electrode but not improve the conductivity of Pd-cells^17^. ACP are likely not affected by this spatial hindrance effect due to their much smaller size. Nevertheless, the current generation in a polymer electrolyte membrane fuel cell with the anode coating with Pd-cells-ACP hybrid was reported to be negligible^28^. One explanation is that the introduced ACP (ACP:Pd was 4:1 w/w) maybe not enough in amount to form a conductive network that well connected the Pd NPs to the electrode. Compared to the ACP, a zero-dimensional material, CNTs as one-dimensional material with the length up to 50 μm are likely to form a conductive network with less amount of material. Thus, even the ratio of CNTs to Pd was lower (1:1 w/w), the activation effect on the Pd NPs was observed in this work. In addition to carbon material, the hybridization with conductive organic polymers, such as polyaniline and polypyrrole, may be also helpful to enhance the catalytic activity of Pd-cells. Previously, polypyrrole was successfully used to improve the electron transfer between electrode and the electrogenic bacteria^32,33^. Since the introduction of conductive polymers can usually increase the stability of the catalysts coating, this approach may provide additional benefit of preventing the possible detachment of Pd-cells and warrant further study.

Electrochemical active surface area of Pd (EASA_Pd_) is an important criteria in Pd related electrochemistry, which in most case positively correlates to the electrocatalytic activity of Pd catalysts^34^. The EASA_Pd_ in the state-of-the-art nano-Pd catalysts prepared by chemical methods were reported from several square meters to dozens of square meters per gram Pd^18,35^. Obviously, the EASA_Pd_ of Pd-cells obtained in this work (0.29 m^2^·g^−1^) is far lower. However, after hybridizing with CNTs, EASA_Pd_ enlarged a lot (to 20.44 m^2^·g^−1^ in Pd-cells-CNTs10) and became comparable to those achieved by using chemical methods. This results indicates bio-Pd could has been an excellent electrochemical catalyst but is just limited by lacking a path for efficient electron transport. With regard to the already reported bio-Pd systems, although they have been used as electro-catalysts for various reactions, few studies reported their EASA_Pd_. In our recently published work, we found EASA_Pd_ in Pd-cells was affected by the ratio of cell dry weight (CDW) to the Pd(II) during the Pd NPs synthesis. At the same CDW:Pd(II) ratio as in this work (1:1), a similar EASA_Pd_ was obtained (0.65 m^2^·g^−1^ vs 0.29 m^2^·g^−1^). The significant increase of EASA_Pd_ was observed when the CDW:Pd(II) ratio was decreased to 1:6. In this case, the formed Pd NPs were much bigger in size (mean size was 54.3 nm and some can reach hundreds nanometer) and therefore could create an electron transport path by connecting each other. However, the increase of NPs size can reduce the real specific surface area of Pd. As a result, the EASA_Pd_ was just measured as 10.6 m^2^·g^−1^, about 2 times lower than that in Pd-cells-CNTs10 with smaller Pd NPs size (centered on 10–30 nm, Fig. 3c).

Besides catalyzing NB reduction, Pd as an efficient electrocatalyst can be also used for other environmental remediation applications, such as reductively degrading many organic halides^15,36,37^, or as an anodic catalyst in fuel cells through oxidizing hydrogen, formic acid, methanol and ethanol^38^. Since the composition of CNTs basically facilitate the electron transport in Pd-cells, the improvement of electrocatalytic activity by this approach is expected to extend to boarder range of Pd-cells associated electrochemical reactions. In addition to bio-Pd, other metal NPs produced by bacteria seems also suffer from the same poor conductive problem because their distribution on the cells are also shown as sporadic^39^. Further study is warranted to verify the effectiveness of introducing conductive materials into these bio-metal systems, which may offer a great opportunity to electrochemically apply these green synthesized nano-materials to more diverse objectives.

## Methods

### Chemicals

Sodium tetrachloropalladate(II) (Na_2_PdCl_4_, 98%), commercial palladium on carbon (Pd/C, 10 wt.% loading), submicron Pd catalyst (Purity > 99 wt%) were purchased from Sigma-Aldrich (Co., USA). MWCNTs (Purity > 95 wt%, -NH2 content 0.45 wt%) with ~50 μm length (8–15 nm outer diameter, 3–5 nm inner diameter) were obtained from Timesnano, China. Other chemicals used were analytical grade at least. All of the aqueous solutions were prepared using Milli-Q water (18.25 MΩ·cm^−1^).

### Microbe preparation

*Shewanella oneidensis* MR-1 were cultured aerobically in fresh LB broth (10 g·L^−1^ peptone, 5 g·L^−1^ yeast extract, 5 g/L NaCl, pH = 7.0) at 30 °C up to mid-logarithmic phase. The cells from these precultures were pelleted by centrifuging at 4500 rpm for 10 min, washed twice, and finally re-suspended to nutrients amended phosphate buffer solution (0.31 g·L^−1^ NH_4_Cl, 0.13 g·L^−1^ KCl, 2.77 g·L^−1^ NaH_2_PO_4_·2H_2_O, 11.55 g·L^−1^ Na_2_HPO_4_ ·12H_2_O, pH = 7.0 ± 0.2). The cell concentration in terms of cell dry weight (CDW) was adjusted to 150 mg·L^−1^.

### Preparation of Pd-cells and Pd-cells-CNTs hybrids

50 mL above-mentioned *Shewanella* cell suspension was transferred to the sterile serum bottles, purged with nitrogen gas (purity > 99.9%) for 20 min, and sealed with butyl rubber stoppers and aluminum caps. Formate and Pd(II), respectively serving as the electron donor and Pd precursor, were supplemented in sequence into the serum bottles, reaching the final concentrations of 25 mM and 150 mg·L^−1^, respectively. The palladized cells (Pd-cells) were then obtained by incubating the mixture in a constant temperature shaker with 120 rpm at 30 °C for 24 hours. Subsequently, the Pd-cells were pelleted by centrifuging at 4500 rpm for 10 min, washed twice, and re-suspended to the fresh PBS for use. To prepare the Pd-cells-CNTs hybrids, MWCNTs were added to the prepared Pd-cells suspensions with the concentration of 150 mg·L^−1^ and 1500 mg·L^−1^. After a well mixing by the Votex, two types of Pd-cells-CNTs hybrids were obtained, which contained low amount of CNTs (the ratio of Pd/ CNTs is 1/1 w/w, Pd-cells-CNTs1) and high amount of CNTs (the ratio of Pd/ CNTs is 1/10 w/w, Pd-cells-CNTs10), respectively. For comparison, the hybrids composed of CNTs (with the same amount as in Pd-cells-CNTs10) and *Shewanella* cells without palladization (cells-CNTs10) were also prepared.

### Electron microscope examinations

For field emission scanning electron microscope (FESEM) examination, the samples were fixed overnight in 2.5% glutaraldehyde amended PBS, dehydrated in a graded series of ethanol (50, 70, 80, 90, and 3 × 100% with 15 min for each level), then sequentially displaced by 50% isoamyl acetate in ethanol and 100% isoamyl acetate for 15 min each, and finally dried in a desiccator for 12 h. Subsequently, the prepared samples were observed by the FESEM (SU8000 Hitachi, Ltd, Japan). Transmission electron microscopy (TEM, JEM-1400 Hitachi, Ltd, Japan) examinations were operated at 100 kV accelerating voltage. The samples were embedded in the Eponate resin, which were sliced into pieces with the thickness of 70 nm. The ultrathin sections were then stained with uranyl acetate and lead citrate in prior to the observation.

### X-ray diffraction characterizations

Pd-cells were coated onto a piece of polycarbonate membrane with pore size of 0.22 μm through filtration and then was dried at room temperature. The samples were then analyzed by an X-ray diffraction (XRD) system (X’Pert PRO MPD, PANalytical, Netherlands). X-rays were generated by a copper X-ray tube with power 1.6 kW (40 kV, 40 mA). Measurements were made between 30° and 90° 2θ with a step size of 0.026° 2θ.

### Electrochemical characterizations

Cyclic voltammetry (CV) and chronoamperometry were conducted by using a potentiostat (1030 C, CH Instruments, China) equipped as the three-electrode system, in which Ag/AgCl electrode (KCl saturated), platinum mesh (1 cm^2^), and glassy carbon electrode (GCE, 4 mm in diameter) were employed as the reference electrode, the counter electrode and the working electrode, respectively. In prior to the measurements, the GCEs were well polished by using 0.3 μm and 0.05 μm alumina slurry and then coated by the Pd-cells, Pd-cells-CNTs, cells-CNTs and commercial Pd catalysts, respectively. For the Pd associated sets, the Pd mass loadings were adjusted identically to 50 ± 3 μg·cm^−2^. while, for the cells-CNTs, the coating was carried out with giving the identical CNTs mass loading as compared to the Pd-cells-CNTs (CDW/CNTs was 1/10, w/w). All above mentioned experiments were performed anaerobically at the temperature of ~26 °C.

In order to get insight into the effect of the CNTs on the conductivity improvement in the hybrids, the split gold electrodes with ~100 nm gap (Zensor R&D, Taiwan) were employed and coated with the Pd-cells and the Pd-cells-CNTs, respectively, with an identical thickness of ~50 μm. A bias voltage was applied across the split electrodes from 0 V to 0.05 V using the potentiostat that was operated as the two-electrode mode with the scan rate of 0.001 V·s^−1^. The recorded currents were plotted as the function of the voltages (I-V curves). The conductance of the coating layer can be obtained directly from the slope of the I-V curves.

### Analysis and calculation

The precise contents of Pd were determined by inductively coupled plasma optical emission spectroscopy (ICP-OES, IRIS 1000, Thermo Elemental Co.). The electrochemically active surface area of Pd (EASA_Pd_ m^2^·g^−1^) was calculated according to the equation (1).

1 $${\rm{E}}{\rm{A}}{\rm{S}}{{\rm{A}}}_{{\rm{P}}{\rm{d}}}{\rm{=}}{\rm{Q}}{\rm{/}}({{\rm{Q}}}_{{\rm{r}}{\rm{e}}{\rm{f}}}{\rm{\times }}{{\rm{P}}{\rm{d}}}_{{\rm{m}}})$$

where Pd_m_ (g) is the Pd mass loaded on the surface of working electrode. Q (C) is the coulombic charges related to the peak of the PdO reduction in CV test with capacitance current removal. Q_ref_ is the constant coulombic (4.05 C·m^−2^) charges associated with the reduction of PdO mono layer on the Pd NPs to element Pd^40^.
